# Translation and Validation of the Nutrition for Sport Knowledge Questionnaire in Brazil (NSKQ-BR)

**DOI:** 10.3390/nu16121891

**Published:** 2024-06-15

**Authors:** Jéssica Bianca Alves de Sousa, Guilherme Falcão Mendes, Renata Puppin Zandonadi, Teresa Helena Macedo da Costa, Bryan Saunders, Caio Eduardo Gonçalves Reis

**Affiliations:** 1 Department of Nutrition, School of Health Sciences, Universidade de Brasília (UnB), Campus Darcy Ribeiro, Asa Norte, Brasilia 70910-900, Brazil; renatapz@unb.br (R.P.Z.); thmdacosta@gmail.com (T.H.M.d.C.); caioedureis@gmail.com (C.E.G.R.); 2 Nutrition and Dietetics Graduate Program, Catholic University of Brasília, Taguatinga, Brasilia 71966-700, Brazil; guile.fm@gmail.com; 3 Applied Physiology and Nutrition Research Group, School of Physical Education and Sport, Faculdade de Medicina FMUSP, Universidade de São Paulo, Sao Paulo 01246-903, Brazil; drbryansaunders@outlook.com; 4 Center of Lifestyle Medicine, Faculdade de Medicina FMUSP, Universidade de São Paulo, Sao Paulo 01246-903, Brazil; 5 Nutrology Academy, Rio de Janeiro 22421-030, Brazil

**Keywords:** surveys and questionnaires, translation and validation, Brazilian, Portuguese, nutritional knowledge, sports

## Abstract

This study aimed to translate, culturally adapt, and validate “The Nutrition for Sport Knowledge Questionnaire (NSKQ)” for Brazilian athletes. The NSKQ is an Australian instrument composed of 87 questions divided into six subsections (weight control, macronutrients, micronutrients, sports nutrition, supplementation, and alcohol) designed to assess the nutritional knowledge (NK) of athletes. The translation process followed the recommendations of the World Health Organization for translating and adapting instruments. Semantic validation involved a panel of specialists (*n* = 21), followed by an assessment performed by a group of adult Brazilian athletes from various sports (*n* = 17). The reproducibility and internal consistency of the questionnaire were evaluated via a test–retest approach in a sample of adult Brazilian athletes (*n* = 29) from diverse sports, who completed the Brazilian version of the NSKQ (NSKQ-BR). Overall, the NSKQ-BR presented good internal consistency (α = 0.95) and reproducibility (intraclass correlation coefficient (ICC) = 0.85). The factors “sports nutrition” and “alcohol” showed moderate reproducibility (ICC = 0.74 (0.46–0.88) and ICC = 0.68 (0.33–0.85), respectively). Most athletes (*n* = 17; 58.6%) presented a medium NK score (50–65%). The NSKQ-BR is available to evaluate the NK levels of Brazilian athletes. The NSKQ-BR presented high internal consistency and reproducibility, validating its applicability among adult athletes across diverse sports.

## 1. Introduction

Dietary intake is crucial for athletes at all levels, from recreational to elite, due to its direct association with the improvement of physiological adaptations to sports training and exercise recovery. However, imbalances in food intake may have unfavorable consequences for health and physical performance. In this regard, the insufficient nutritional knowledge (NK) of athletes may be a barrier to adequate dietary intake [1,2]. Therefore, nutrition education interventions may be beneficial to improving NK and supporting positive dietary changes [3,4].

NK questionnaires are effective tools to measure an athlete’s NK level and to evaluate the effectiveness of nutrition education interventions [5]. Concerning this, a systematic review emphasized that all available questionnaires used to evaluate NK among athletes have limitations, including outdated guidelines, inadequate validation processes, and limited cultural adaptation. Therefore, performing the assessment of NK levels in athletes presented a risk of bias due to the fragile context [6]. Subsequently, many better-quality NK questionnaires have been developed to address the aforementioned limitations [3,5].

The Nutrition for Sport Knowledge Questionnaire (NSKQ) was developed to assess the level of sport NK. The questionnaire was validated using a robust methodology and current international recommendations on sports nutrition, and applied to several sports [7]. Furthermore, the NSKQ has also been translated into five languages, namely German, Swedish, Turkish, Chinese, and Malay [8].

In Brazil, there is currently no validated questionnaire to assess the NK of Brazilians. Most studies have used adaptations of questionnaires from other populations or used non-validated instruments [9,10,11,12,13]. The lack of methodological rigor and absence of sport-related inquiries elevate the potential for bias in the obtained results. To this end, a validated and adapted international questionnaire in Brazilian Portuguese could enhance the assessment of NK among Brazilian athletes, providing reliable data and enabling meaningful cross-country comparisons. In addition, the instrument will support future research on sport NK and assist dietitians and physicians in clinical practice. Therefore, this study aimed to translate, culturally adapt, and validate the NSKQ to the Brazilian athlete population.

## 2. Materials and Methods

This cross-sectional study was approved by the Research Ethics Committee of the Faculty of Health Sciences of the University of Brasília, Brazil (CAAE 58628722.3.0000.0030), on 27 July 2022 and was conducted in three stages: (i) translation; (ii) semantic assessment and cultural adaptation; and (iii) assessment of internal consistency and reproducibility.

The present study used the Australian NSKQ (original in English) developed by Trakman et al. (2017) [7] with adjustments made in 2019 regarding the food nutrient content, protein recommendations, and the legality of supplements [8]. The questionnaire has 87 items divided into six subsections: weight control, macronutrients, micronutrients, sports nutrition, supplementation, and alcohol. The answer formats include multiple choice questions, “agree/disagree/not sure”, and “effective/not effective/not sure”, with 1 point assigned for each correct answer. The NK quantification is given by the percentage-based scoring system established by the original NSKQ´s authors, as follows: “poor” (0–49% right), “medium” (50–65%), “good” (66–75%), and “excellent” (76–100%).

### 2.1. Translation

The process followed the recommendations of the World Health Organization for the translation and adaptation of instruments [14]. Following contact and authorization from the responsible author, the original version of the questionnaire (NSKQ in English) was translated into the Brazilian Portuguese language by a bilingual researcher native to Portuguese and familiar with the sports nutrition field.

Subsequently, back-translation (from Brazilian Portuguese to English) was performed by a bilingual researcher who was native to England and had resided in Brazil for ten years, with no prior knowledge of the questionnaire and also familiar with the sports nutrition field.

Thereafter, a panel of three bilingual researchers (native to Portuguese) with expertise in the sports nutrition field analyzed the back-translated version (in English) using the original questionnaire (English-to-English comparison). Then, they analyzed the translated version (in Brazilian Portuguese) to solve any possible non-conformities.

After necessary corrections, the final version of the questionnaire was revised by the same two translator researchers (native in Portuguese and native in English) to finalize the translation of the questionnaire into the Brazilian Portuguese language, “The Nutrition for Sport Knowledge Questionnaire—Brazil” (NSKQ-BR). The translation process was performed at the middle school level for a broad understanding of the athlete population [15,16].

The imperial system used in the NSKQ (kilojoules, ounces, pounds, and mmol/L) was excluded, leaving only the metric system, which is used in Brazil (kilocalories, grams, kilograms, and mL/dL). In addition, the unusual foods consumed in Brazilian culture were changed for habitual options with a similar nutritional composition and purpose (nutrient targeted by the question), e.g., Question 2.9.3. “1 cup baked beans” was changed to “1 full ladle baked beans” and Question 2.9.4. “1/2 cup cooked quinoa”, which contains 4 g of protein, was changed to “3 serving spoons boiled rice”, presenting the same 4 g of protein. For this, the Brazilian Food Composition Table (TBCA 7.2) was utilized for food substitutions, and the GloboDiet manual (a photographic manual for food quantification) was used for home measurement adjustments [16,17].

### 2.2. Semantic Assessment and Cultural Adaptation

This step was performed by two panels of judges in two different stages to assess the clarity of the questionnaire (semantic aspect and cultural adaptation). First, a panel of 21 health professionals with master’s or doctorate degrees and sports nutrition experience evaluated the clarity of the questionnaire (panel of specialists). After approval by specialists, a panel of 17 healthy Brazilian athletes (aged between 18–59 years) also evaluated the clarity of the questionnaire (panel of athletes) [18]. For athletes, the exclusion criteria included being a nutrition student or a nutritionist. The judges (specialists and athletes) were enrolled in the study through a social media advertisement (Instagram and WhatsApp by Meta, Inc. Menlo Park, CA, USA) and via direct contact with coaches.

For semantic assessment and cultural adaptation, the NSKQ-BR was inserted into the SurveyMonkey© platform version 4.2.0 (SurveyMonkey Inc., San Mateo, CA, USA). After they provided consent to participate in the study and answered questions regarding their sociodemographic status (age, gender, region, education level, sport and athletic experience), the judges individually analyzed the clarity parameters of each question (*n* = 87) on a Likert scale ranging from 0 to 5, where 0 indicates “I didn’t understand anything”; 1—“I understand just a little”; 2—“I understand more or less”; 3—“I understood almost everything, but I had some doubts”; 4—“I understood almost everything”; and 5—“ I understood perfectly and had no doubts”. The judges could make suggestions regarding any question to improve the clarity of the questionnaire.

To assess the degree of agreement between judges, Kendall’s coefficient of agreement (W) was used. In case of disapproval (W values < 0.8 with Likert score < 4), the item was rewritten according to the suggestions provided and reassessed by the two panels of judges until the required degree of agreement was obtained (W ≥ 0.8 with Likert score ≥ 4) [19,20].

After the semantic evaluation and cultural adaptation stages, the questionnaire was reviewed by the same three researchers who participated in the translation stage, resulting in the final version of NSKQ-BR.

### 2.3. Assessment of Internal Consistency and Reproducibility

To assess the internal consistency and reproducibility of NSKQ-BR, a total of 29 Brazilian athletes aged 18–59 years completed the questionnaire [21]. For this purpose, athletes were asked to complete the NSKQ-BR questionnaire on the Survey-Monkey© platform version 4.2.0 (SurveyMonkey Inc. San Mateo, CA, USA), which included a consent form and socio-demographic questions (age, gender, region, education level, sport and athletic experience). The volunteer athletes were invited to participate in the study by contacting their coaches and through social media advertisements (Instagram and WhatsApp by Meta, Inc. Menlo Park, CA, USA). The exclusion criteria for this stage included not being a nutrition student or a nutritionist. The questionnaire was applied twice (test-retest) without prior knowledge of the second application. Therefore, the questionnaire was sent 48 h after the first application and needed to be answered within 7 days [22].

### 2.4. Statistical Analysis

Sociodemographic data are presented using the absolute frequency and mean with standard deviation (for normal distribution) or median with interquartile range (for non-normal distribution). The Shapiro–Wilk test was used to assess normality and the Sturges rule was applied to determine the number of classes [19]. Kendall’s coefficient of agreement (W), which varies from 0 to 1, was used to assess the questionnaire translation process through semantic evaluation and cultural adaptation. Values of W ≥ 0.8 indicate convergence in the analysis, while W < 0.8 demonstrates disagreement between respondents [20,22]. To determine the reproducibility of the questionnaire, the intraclass correlation coefficient (ICC) was applied to perform the test–retest comparison analysis. Cronbach’s alpha coefficient (α) was used to verify the internal consistency of the questionnaire. Values of ICC > 0.75 and α > 0.7 were considered concordant and significant (*p* < 0.05) [23,24]. Pearson’s correlation (or Spearman’s rank correlation) was performed to assess the correlation between the test–retest NK score. In addition, Spearman’s rank correlation coefficient or Fisher’s exact test was applied to analyze the correlation between sociodemographic data and the NK score. All statistical analyses were performed using IBM SPSS (Statistical Package for Social Sciences) version 22 (IBM SPSS Statistics for Windows, IBM Corp, Armonk, NY, USA).

## 3. Results

**Figure 1:**
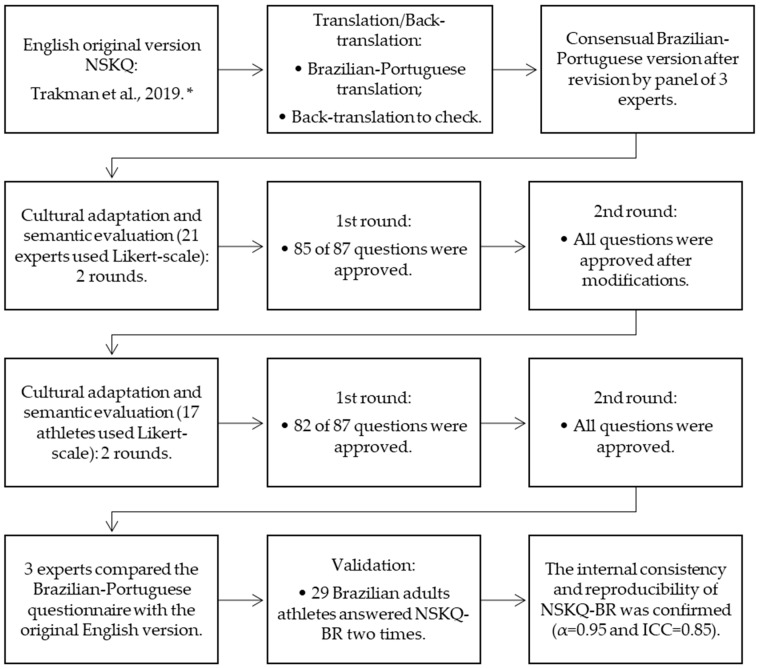
Flowchart of translation, cultural adaptation, semantic evaluation and consistency validation stages of NSKQ-BR. * [8].

The NSKQ-BR (Supplementary File) was developed through the translation, cultural adaptation, semantic assessment, internal consistency, and reproducibility evaluation stages. A summary of this process is shown in Figure 1.

Regarding the semantic assessment and cultural adaptation, the panel of specialists (*n* = 21; 37 ± 5 y; 66.7% with a doctorate) assessed the clarity of the questionnaire. In the first round, two questions were reproved (1.3.4 and 2.1; W = 0.74 and 0.78 with Likert score < 4, respectively). Following the suggested revisions by the specialists, the questionnaire achieved the required agreement for the 87 items (W = 0.96 with a Likert score ≥ 4). In the second stage of the clarity evaluation, a panel of 17 athletes (36 ± 9 y; 6 ± 13 y of athletic experience) conducted two rounds of assessments. Adjustments were performed on five questions (2.2.3, 2.6, 4.10, 6.1, and 6.2), with athletes reaching agreement on the evaluation of the 87 items in the questionnaire (W = 0.82 with a Likert score ≥ 4). The characteristics of the specialists and athletes are shown in Table 1.

Concerning the assessment of internal consistency and reproducibility, 29 Brazilian athletes from different sports (39 ± 10 y; 8 ± 7 y of athletic experience) participated in the test–retest stage. The average response time during the test was 21 min, while for the retest, it was 17 min. Among the athletes, 24 (86.2%) had received nutritional counseling at some point, of which 14 (48.3%) were still under such counseling at the time of the study. In terms of the primary source of nutritional information, 17 (58.6%) mentioned nutritionists, 7 (24.1%) cited health professionals (such as doctors, trainers, and physiotherapists), and 5 (17.2%) referred to social media. The characteristics of the athletes are shown in Table 2.

Overall, the NSKQ-BR presented a good internal consistency (α = 0.95) and reproducibility (ICC = 0.85). However, as shown in Table 3, when analyzed individually, the factors “sports nutrition” and “alcohol” showed only moderate reliability (reproducibility) (ICC = 0.74 (0.46–0.88) and ICC = 0.68 (0.33–0.85), respectively) [24]. The overall NK score was 59.1 ± 9.3% at test and 56.9 ± 12.8% at retest, with a strong positive correlation found between the test and retest total scores (Pearson’s r = 0.76; *p* = <0.001). Moderate positive correlations were shown for the factors “macronutrients” (*r* = 0.65), “sports nutrition” (*r* = 0.61), and “alcohol” (*r* = 0.51) (all *p* = 0.01).

**Figure 2:**
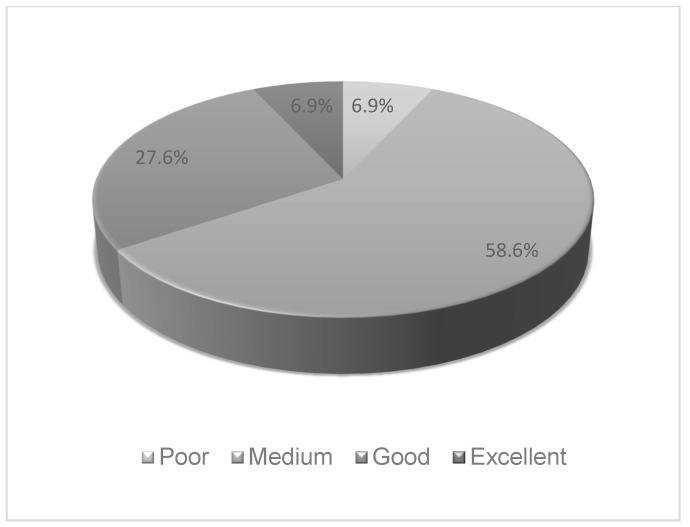
Pie chart of Nutritional Knowledge assessment (*n* = 29). Poor (0–49% correct); Medium (50–65%); Good (66–75%); Excellent (76–100%).

The mean NK score observed among the athletes assessed (*n* = 29) was 59.1%. Most of the athletes (*n* = 17; 58.6%) presented a medium NK score (Figure 2). Of those who obtained a high score (good (66–75%) and excellent (76–100%)), 80.0% (*n* = 8) were men, had a high level of education (bachelor’s degree or more) and received nutritional monitoring, 60.0% (*n* = 6) practiced endurance sports, and 40.0% (*n* = 4) had 7 to 13 years of athletic experience and obtained nutritional information from a nutritionist. Among those with low scores (poor (<50%) and medium (50–65%)), 52.6% (*n* = 10) were men, 84.2% (*n* = 16) had a high level of education, 68.4% (*n* = 13) practiced endurance sports, 47.4% (*n* = 9) had 1 to 7 years of athletic experience, 89.5% received nutritional monitoring (*n* = 17), and 68.4% (*n* = 13) obtained nutritional information from a nutritionist. However, no correlation was found between the sociodemographic data and NK score (Table 4).

## 4. Discussion

This is the first study to translate and validate an NK questionnaire regarding sports nutrition for Brazilian athletes. Until now, no properly constructed and validated questionnaire that is available in the Brazilian Portuguese language has been adapted to the dietary context of the Brazilian population. The NSKQ-BR was designed to assess athletes’ knowledge of nutritional aspects (macronutrients and micronutrients), weight control, supplementation, sports nutrition, and alcohol.

In general, the questionnaire presented good reliability and reproducibility in the overall score (α = 0.95; ICC = 0.85). In the factors analysis, only “sports nutrition” and “alcohol” did not achieve adequate reproducibility, presenting a marginal value (ICC = 0.74 and ICC = 0.68, respectively). However, a similar result was observed in the original NSKQ, where authors found adequate reliability and reproducibility in the global aspect (KR-20 = 0.87 and Pearson’s r = 0.92 (*p* < 0.001)) but inadequate values for “alcohol” (KR-20 = 0.5) and “supplementation” (Pearson’s r = 0.6) [7]. Therefore, while the general response to the questionnaire when applied in future work can be considered reliable and reproducible, caution must be taken regarding the specific responses to sports nutrition and alcohol.

The mean NK score was lower in all domains in the second application of the NSKQ-BR, except for the “alcohol” factor, which presented similar values (test: 50.0 ± 12.5; retest: 50.0 ± 25.0). In the retest, the “alcohol” factor showed high variation in responses, which could have affected its reproducibility value, as the ICC is sensitive to high variability between individuals [24]. Therefore, this probably occurred due to participants guessing the answers.

The mean NK score observed among the athletes assessed was 59.1%, indicating a medium level of knowledge. Comparable findings were reported among elite English squash players (56.1%) and professional athletes in the Australian women’s football league (50.6%) [26,27]. Conversely, studies conducted with professional Irish Gaelic footballers, Australian team sports athletes, and Scottish rugby athletes revealed a poor NK level (<50%) [28,29,30,31]. Similar to the current study, no correlation was demonstrated between age, educational level and the level of NK in the athletes [26,27]. In the present study, athletes demonstrated high levels of knowledge (>65%) about “weight management” and “macronutrients” factors. These outcomes can inform more targeted and effective nutritional education initiatives. In this context, the use of NK questionnaires in sports practice can help to optimize the performance of coaches and sports nutritionists to combat misinformation among athletes.

Inadequate NK in athletes may be responsible for insufficient food consumption [1]. In Brazilian athletes, estimates suggest a prevalence of low energy availability (LEA) ranging from 62% to 88% [32]. Inadequate dietary intake, a factor that contributes to LEA, may be addressed by athletes through increasing their NK levels. In this regard, the evaluation of NK enables the identification of gaps in athletes’ knowledge, which supports the creation of effective educational programs [2,4].

In this context, the NSKQ-BR is a reliable tool to assess NK, and will improve the clinical practice of coaches and sports nutritionists to prevent and treat the inadequate dietary intake of Brazilian athletes aiming to optimize exercise performance. Furthermore, we expect that the NSKQ-BR will help athletes translate the NK into better food consumption patterns, thereby improving recovery and sports performance.

The use of a validated and adapted international questionnaire allows the reliable comparison of data across different countries [6]. Similar to the current study, other authors successfully adapted the NSKQ for use in Turkey. Following cultural adjustments, the questionnaire demonstrated a high degree of validity and reliability (α = 0.908; Pearson’s r > 0.5) [33]. However, the study was published in the Turkish language, which precludes a full understanding of the manuscript. Furthermore, among all the languages into which the NSKQ was translated (German, Swedish, Turkish, Chinese, and Malay) [8], only the Turkish version has been published as a scientific article [33]. Therefore, future studies proposing to translate the NSKQ should follow the international guidelines for translation and validation to obtain a validated questionnaire and generate reliable results.

Although web-based research may have limitations, recent official data indicate that the majority of the Brazilian population (87.2%) has access to the internet [34]. The Brazilian Portuguese language presents heterogeneity due to the vast extent of its territory. In this regard, the online application enabled Brazilian regions to be better represented. Furthermore, the sample consisting of athletes from diverse educational levels, sports, and athletic experiences supports the applicability of the NSKQ-BR in various Brazilian sporting contexts.

The length of time required to complete the questionnaire (17 to 21 min) may serve as a barrier for specific groups of athletes or nutritional goals. Consequently, future studies are required to develop an abbreviated version of the NSKQ-BR to improve its application in several contexts. In addition, the moderate reproducibility observed in the “sports nutrition” and “alcohol” factors is a limitation of the presented study, even as the original NSKQ. Therefore, although the original NSKQ allows the independent application of factors [7], the NSKQ-BR does not allow the robust application of the “sports nutrition” and “alcohol” factors due to the fragility of the reproducibility (ICC = 0.74 and ICC = 0.68, respectively). Overall, the NSKQ-BR´s translation and validation for Portuguese and Brazilian culture were successful.

## 5. Conclusions

“The Nutrition for Sport Knowledge Questionnaire—Brazil” is now available to evaluate the NK levels of Brazilian athletes. The NSKQ-BR presented high internal consistency and reproducibility, validating its applicability among adult athletes across diverse sports. Notably, the “sports nutrition” and “alcohol” factors exhibited moderate reproducibility only, suggesting that caution should be exercised when performing the isolated assessment of these specific factors. This study improves the assessment of NK in sports, enabling more effective nutritional strategies for Brazilian athletes and sports nutritionists. Future research using the NSKQ-BR to assess the NK of Brazilian athletes is expected, and adjustments could be applied in advance to sports nutrition recommendations.

## Figures and Tables

**Table 1:** Socio-demographic data and sample profile of specialists (*n* = 21) and athletes (*n* = 17) included in the semantic evaluation.

Specialists (*n* = 21)		*N*	(*%* Available Data)
Age (in years) Gender			36.8 (5.5) ^a^
	Female	12	57.1
	Male	9	42.9
Region *	Midwest	11	52.4
	Southeast	9	42.8
	Northeast	1	4.8
Degree of expertise	Nutrition	16	76.2
	Nutrition and Physical Education	3	14.3
	Physical Education	2	9.5
Educational Level	Doctorate	14	66.7
	Master	7	33.3
Athletes (*n* = 17)		*N*	(% available data)
Age (in years)			36.2 (9.1) ^a^
Gender	Male	13	76.5
	Female	4	23.5
Region *	Midwest	13	76.4
	Southeast	2	11.8
	Northeast	2	11.8
	Postgraduate	7	41.2
Educational Level	Bachelor’s degree	5	29.4
	High school	4	23.5
	Elementary school	1	5.9
Sport	Endurance	11	64.7
	Strength	4	23.5
	Miscellaneous **	2	11.8
Athletic Experience (in years)	2–7	9	52.9
	8–13	3	17.6
	14–19	3	17.6
	20–25	1	5.9
	26–32	1	5.9

^a^ Mean and standard deviation; * Human development by Brazilian region (2016): Midwest—0.757; Southeast—0.766; Northeast—0.663 [25]; ** Judo and Soccer.

**Table 2:** Socio-demographic data of athletes included in the test–retest (*n* = 29).

		N	(*%* Available Data)
Age (in years)			38.5 (10.2) ^a^
Gender	Male	18	62.1
	Female	11	37.9
Region **	Midwest	16	55.2
	Southeast	9	31.0
	Northeast	2	6.9
	South	2	6.9
	Bachelor´s degree	13	44.8
Educational Level	Postgraduate studies	11	37.9
	High school	3	10.3
	Elementary school	2	6.9
Sport	Endurance	19	65.5
	Strength	7	24.1
	Combat sports	3	10.3
Athletic Experience (in years)	1–6	12	41.4
	7–12	12	41.4
	13–18	3	10.3
	19–24	1	3.4
	25–30	0	0.0
	31–37	1	3.4

^a^ Mean and standard deviation; ** Human development by Brazilian region (2016): Midwest—0.757; Southeast—0.766; Northeast—0.663; South—0.754 [25].

**Table 3:** Test-retest data (*n* = 29).

Factor	Test (%)	Retest (%) ^a^	α (95% CI)	ICC (95% CI) ¶	Correlation Coefficient	*p*-Value
Weight Management	75.0 ± 25.0 *	69.3 ± 13.9	0.74 (0.62–0.87)	0.88 (0.74–0.94)	0.77	0.01
Macronutrients	68.7 ± 10.6	66.9 ± 12.4	0.89 (0.82–0.94)	0.78 (0.54–0.90)	0.65	0.01
Micronutrients	50.4 ± 18.3	48.5 ± 21.5	0.90 (0.84–0.95)	0.86 (0.70–0.93)	0.76	0.01
Sports Nutrition	55.8 ± 15.9	52.3 ± 20.3	0.78 (0.64–0.88)	0.74 (0.46–0.88)	0.61	0.01
Supplementation	48.3 ± 22.5	41.7 ± 23.8 *	0.78 (0.65–0.88)	0.83 (0.63–0.92)	0.71	0.01
Alcohol	50.0 ± 12.5 *	50.0 ± 25.0 *	0.79 (0.66–0.89)	0.68 (0.33–0.85)	0.51	0.01
Overall	59.1 ± 9.3	56.9 ± 12.6	0.95 (0.93–0.98)	0.85 (0.68–0.93)	0.76	0.01

^a^ Interval average of test–retest: 5 ± 2 days; * Nonparametric distribution; ¶ Two-way mixed effects model, absolute agreement.

**Table 4:** Nutritional Knowledge score by sample profile of athletes (*n* = 29).

		Poor/Medium *n* (%)	Good/Excelent *n* (%)	*p*-Value
Gender	Male	10 (52.6%)	8 (80.0%)	*p* = 0.234
	Female	9 (47.4%)	2 (20.0%)	
Educational Level	Low *	3 (15.8%)	2 (20.0%)	*p* = 0.576
	High **	16 (84.2%)	8 (80.0%)	
Sport	Endurance	13 (68.4%)	6 (60.0%)	
	Strength	4 (21.1%)	3 (30.0%)	*p* = 0.865
	Combat sports	2 (10.5%)	1 (10.0%)	
Athletic Experience (in years)	1–6	9 (47.4%)	3 (30.0%)	
	7–12	6 (31.6%)	4 (40.0%)	
	13–18	3 (15.8%)	2 (20.0%)	*p* = 0.540
	19–24	0 (0.0%)	1 (10.0%)	
	25–30	0 (0.0%)	0 (0.0%)	
	31–37	1 (5.3%)	0 (0.0%)	
Nutritional Monitoring	Yes	17 (89.5%)	8 (80.0%)	*p* = 0.592
	No	2 (10.5%)	2 (20.0%)	
Nutritional Information	Nutritionist	13 (68.4%)	4 (40.0%)	*p* = 0.278
	Health professional Social media	4 (21.1%) 2 (10.5%)	3 (30.0%) 3 (30.0%)	

* Elementary school and High school; ** bachelor’s degree and postgraduate studies.

## Data Availability

The data presented in this study are available on request from the corresponding author due to ethical reasons.

## Supplementary Materials

The following supporting information can be downloaded at: https://www.mdpi.com/article/10.3390/nu16121891/s1.
