# Supplementary material for: Translation and Validation of the Nutrition for Sport Knowledge Questionnaire in Brazil (NSKQ-BR)
Source: Nutrients. 2024 Jun 15;16(12):1891. doi: 10.3390/nu16121891 (PMC11206272; doi:10.3390/nu16121891)
Supplement: Supplementary file 1 [file nutrients-16-01891-s001.zip › nutrients-3043540-supplementary.pdf]

nutrients

MDPI

Questionário de Conhecimento Nutricional para o Esporte, versão brasileira (NSKQ - BR).

Controle de Peso

Q1.1 Qual nutriente você acha que tem mais calorias por 100 gramas?

- ❍ Carboidrato
- ❍ Proteína
- ❍ **Gordura**
- ❍ Não sei

Q1.2 Você concorda ou discorda com os itens abaixo sobre perda de peso?

	Concordo	Discordo	Não sei
1. Em exercícios de longa duração (ex.: ciclismo, triatlo e corrida), ter o menor peso possível beneficia o desempenho a longo prazo **(D)**	❍	❍	❍
2. Comer mais proteína é o mais importante se você quiser ter mais músculo **(D)**	❍	❍	❍
3. Comer mais proteínas do que você precisa pode fazer você engordar **(C)**	❍	❍	❍

Q1.3 Você acha que as mudanças na alimentação descritas abaixo são boas maneiras de perder peso?

	Sim	Não	Não sei
1. Trocar carboidratos ou alimentos mais calóricos por alimentos de baixa caloria, como vegetais **(S)**	❍	❍	❍
2. Comer margarina em vez de manteiga **(N)**	❍	❍	❍
3. Comer barras de proteína e shakes em vez de iogurte, granola e frutas **(N)**	❍	❍	❍
4. Escolher carboidratos de baixo índice glicêmico (ex: arroz e pão integrais, aveia, batata doce, etc) para ajudar a regular o apetite **(S)**	❍	❍	❍

Q1.4 Para perder peso, os atletas devem:

- ❍ comer menos de 50 gramas de carboidrato por dia
- ❍ comer menos de 20 gramas de gordura por dia
- ❍ **comer menos calorias que ele precisa**
- ❍ Não sei

Q1.5 Para garantir que atendam às suas necessidades de caloria, os atletas devem:

- ❍ **planejar sua dieta com base na idade, gênero, peso e altura, tipo de esporte e programa de treinamento**
- ❍ comer com base em seus sinais de fome e saciedade
- ❍ comer pelo menos 2.000 calorias por dia
- ❍ comer mais alimentos que têm muito carboidrato
- ❍ Não sei

Q1.6 Qual é a melhor opção de refeição pós-treino para um atleta que quer ganhar massa muscular?

- ❍ Um *shake* hipercalórico e hiperproteico e 3 - 4 ovos mexidos
- ❍ **Macarrão ao molho com carne magra e vegetais, além de sobremesa de frutas, iogurte e castanhas**
- ❍ Um pedaço grande de frango grelhado com salada (ex.: alface, pepino, tomate)
- ❍ Um bife grande e ovos fritos
- ❍ Não sei

Q1.7 Qual é a melhor opção de refeição pós-treino para um atleta que deseja perder peso?

- ❍ Uma salada sem molho (ex.: alface, pepino, tomate)
- ❍ *Whey protein* isolado misturado com água
- ❍ **Refeição que inclui: uma porção pequena a moderada de carboidrato com carne (ex.: um prato raso de macarrão ao molho com carne moída magra e vegetais) e uma salada grande**
- ❍ Não sei

Macronutrientes

Q2.1 Um atleta de exercícios de longa duração (ex.: ciclismo, triatlo e corrida), que treina cerca de duas horas por dia de moderada a alta intensidade, deve comer:

- ❍ 1 a 3 gramas de carboidratos por quilo de peso corporal por dia
- ❍ **5 a 7 gramas de carboidratos por quilo de peso, chegando até 10 gramas por quilo de peso quando o treino for intenso ou em competição**
- ❍ 75 - 85% da ingestão diária de calorias vindas dos carboidratos
- ❍ Não sei

Q2.2 Observe as opções a seguir e avalie se elas possuem carboidratos suficientes para se recuperar de um treino aeróbico de alta intensidade com cerca de 1 hora. Suponha que o atleta pese cerca de 70kg e tenha um treino importante novamente amanhã.

	Suficiente	Insuficiente	Não sei
1. banana média (75g) **(I)**	❍	❍	❍
2. 3 colheres de servir de macarrão cozido (150g) e 1 lata de atum (120g) **(I)**	❍	❍	❍
3. 1 pote de iogurte natural (170g) **(I)**	❍	❍	❍
4. 2 colheres de servir de arroz (110g) e 1 concha cheia de feijão (140g) **(S)**	❍	❍	❍

Q2.3 Qual alimento tem mais carboidrato?

- ❍ **3 colheres de servir de arroz cozido (165g)**
- ❍ 2 fatias de pão de forma branco (50g)
- ❍ 1 batata média cozida (150g)
- ❍ 2 bananas maduras médias (150g)
- ❍ Não sei

Q2.4 Você concorda ou discorda destas afirmações sobre gordura?

	Concordo	Discordo	Não sei
1. O corpo precisa de gordura para prevenir doenças **(C)**	❍	❍	❍
2. Atletas não devem comer mais de 20g de gordura por dia **(D)**	❍	❍	❍
3. Quando aumentamos a intensidade do exercício, usamos mais gordura como combustível **(D)**	❍	❍	❍
4. Quando nos exercitamos em baixa intensidade, nosso corpo usa principalmente gordura como combustível **(C)**	❍	❍	❍

Q2.5 Você acha que esses alimentos são ricos em gordura

	Sim	Não	Não sei
Queijo cheddar **(S)**	❍	❍	❍
Margarina **(S)**	❍	❍	❍
Mix de castanhas **(S)**	❍	❍	❍
Mel **(N)**	❍	❍	❍

Q2.6 Você concorda ou discorda destas afirmações sobre proteína?

	Concordo	Discordo	Não sei
1. A proteína é o principal combustível que os músculos usam durante o exercício **(D)**	❍	❍	❍
2. Atletas vegetarianos podem atender às suas necessidades de proteína sem o uso de suplementos proteicos **(C)**	❍	❍	❍
3. Um atleta experiente precisa de mais proteína que um atleta que está apenas começando a treinar **(D)**	❍	❍	❍
4 O corpo tem uma capacidade limitada de usar proteínas para formar de músculos **(C)**	❍	❍	❍
5. Uma dieta equilibrada, com calorias (energia) suficientes, tem proteína suficiente para a maioria dos atletas **(C)**	❍	❍	❍

Q2.7 Qual alimento tem mais proteína?

- ❍ 2 ovos cozidos
- ❍ **100g de peito de frango sem pele cozido (1 filé médio)**
- ❍ 30g de amêndoas (25 unidades)
- ❍ Não sei

Q2.8 Um atleta experiente de 100 kg que faz treino de força (ex.: musculação) precisa consumir qual quantidade de proteína entre as opções abaixo:

- ❍ 100g (1g/kg de peso)
- ❍ **150g (1.5g/kg de peso)**
- ❍ 500g (5g/kg de peso)
- ❍ Ele deve comer o máximo de proteína que conseguir
- ❍ Não sei

Q2.9 Qual destes alimentos você acha que tem proteína suficiente para aumentar a massa muscular após um exercício de força (ex.: musculação)?

	Suficiente	Insuficiente	Não sei
1. 100g de peito de frango (1 filé médio) **(S)**	❍	❍	❍
2. 300g de queijo prato (15 fatias) **(I)**	❍	❍	❍
3. 140g de feijão cozido (1 concha cheia) **(I)**	❍	❍	❍
4. 160g de arroz branco cozido (3 colheres de servir) **(I)**	❍	❍	❍

Q2.10 Você acha que esses alimentos têm todos os aminoácidos essenciais em quantidade suficiente para o nosso corpo?

	Sim	Não	Não sei
Bife **(S)**	❍	❍	❍
Ovos **(S)**	❍	❍	❍
Feijão **(N)**	❍	❍	❍
Leite de vaca **(S)**	❍	❍	❍

Q2.11 A quantidade de proteína no leite desnatado em comparação ao leite integral é:

- ❍ muito menor
- ❍ **praticamente igual**
- ❍ muito menor
- ❍ Não sei

Micronutrientes

Q3.1 Você concorda ou discorda destas afirmações sobre vitaminas e minerais?

	Concordo	Discordo	Não sei
1. O cálcio é o principal componente do osso **(C)**	❍	❍	❍
2. A vitamina C é um antioxidante **(C)**	❍	❍	❍
3. A tiamina (vitamina B1) é necessária para levar oxigênio aos músculos **(D)**	❍	❍	❍
4. O ferro é necessário para produzir energia a partir do alimento **(D)**<br>5. Vitamina D aumenta a absorção de cálcio **(C)**<br>6. Carne bovina, frango e peixe são boas fontes de zinco **(C)**<br>7. Grãos integrais são boas fontes de vitamina C **(D)**<br>8. Frutas e vegetais são boas fontes de cálcio **(D)**<br>9. Peixe rico em gordura é uma boa fonte de vitamina D **(C)**<br>10. Mulheres que menstruam mensalmente precisam de mais ferro que homens **(C)**<br>11. Atletas de 15 a 24 anos precisam de 500 mg de cálcio por dia **(D)**<br>12. Uma pessoa em forma, com uma dieta equilibrada, pode melhorar seu desempenho físico comendo mais alimentos ricos em vitaminas e minerais **(D)**<br>13. Vitaminas são nutrientes que contêm energia (calorias) **(D)**	❍	❍	❍

Nutrição Esportiva

Q4.1 Os atletas devem beber água para:

- ❍ **manter o volume do sangue estável**
- ❍ parar com a sensação de boca seca
- ❍ suar de forma adequada
- ❍ todos os itens anteriores
- ❍ Não sei

Q4.2 Especialistas sugerem que os atletas devem:

- ❍ beber 50 - 100 ml de líquidos a cada 15 - 20 minutos
- ❍ chupar cubos de gelo em vez de beber água durante o exercício
- ❍ tomar bebidas esportivas (por exemplo, Gatorade) em vez de água ao se exercitar
- ❍ **beber líquidos de acordo com um plano, com base nas mudanças de peso corporal durante sessões de treinamento realizadas em um clima semelhante**
- ❍ Não sei

Q4.3 Durante o exercício, qual a quantidade de sódio que os líquidos para fins de hidratação devem conter?
- ❍ <u>**Pelo menos 250 - 575 mg de sódio por litro**</u>
- ❍ Pelo menos 90 - 185 mg de sódio por litro
- ❍ Nenhum
- ❍ Não sei

Q4.4 Antes da competição, os atletas devem comer alimentos ricos em:
- ❍ líquidos, gorduras e carboidratos
- ❍ líquidos, fibras e carboidratos
- ❍ <u>**líquidos e carboidratos**</u>
- ❍ Não sei

Q4.5 Você concorda com estas afirmações sobre carboidratos?

	Concordo	Discordo	Não sei
1. Comer carboidratos quando você se exercita torna mais difícil construir músculos e ganhar força <u>**(D)**</u>	❍	❍	❍
2. Em eventos com duração de 60 a 90 minutos, 30 a 60 g de carboidratos devem ser consumidos por hora <u>**(C)**</u>	❍	❍	❍
3. Comer carboidratos quando você se exercita ajudará a manter os níveis de açúcar no sangue estáveis <u>**(C)**</u>	❍	❍	❍

Q4.6 Some athletes get a sore stomach if they eat during exercise. What might make stomach pain worse?
- ❍ <u>**Consumir géis energéticos em vez de água ou bebidas esportivas**</u>
- ❍ Consumir pequenas quantidades de água a cada vez
- ❍ Consumir bebidas esportivas com diferentes tipos de carboidratos (por exemplo, frutose e sacarose)
- ❍ Não sei

Q4.7 Durante uma competição, os atletas devem comer alimentos ricos em:
- ❍ Líquidos, fibras e gorduras
- ❍ Líquidos e proteínas
- ❍ <u>**Líquidos e carboidratos**</u>
- ❍ Não sei

Q4.8 Qual é o melhor lanche para consumir durante uma sessão de treino intenso de 90 minutos?
- ❍ Um *shake* de proteína
- ❍ <u>**Uma banana madura**</u>
- ❍ 2 ovos cozidos
- ❍ Um punhado de nozes
- ❍ Não sei

Q4.9 Após uma competição, os atletas devem comer alimentos ricos em:

- ❍ Proteína, carboidrato e gordura
- ❍ Apenas proteína
- ❍ Apenas carboidrato
- ❍ **Carboidratos e proteínas**
- ❍ Não sei

Q4.10 Quanto de proteína você acha que é recomendado aos atletas consumirem após um exercício de força (ex.: musculação)?

- ❍ **0,3 g/kg de peso corporal (cerca de 15 - 25 g de proteína)**
- ❍ 1,0 g/kg de peso corporal (cerca de 50 - 100 g de proteína)
- ❍ 1,5 g/kg de peso corporal (cerca de 150 – 300 g de proteína)
- ❍ Não sei

Suplementação

Q5.1 Você concorda com estas afirmações sobre suplementos vitamínicos e minerais?

	Concordo	Discordo	Não sei
1. A vitamina C deve sempre ser consumida por atletas **(D)**	❍	❍	❍
2. Vitaminas do complexo B devem ser consumidas quando estiver me sentindo fraco **(D)**	❍	❍	❍
3. Comprimidos de sal devem ser consumidos por atletas que têm cãibras quando se exercitam **(D)**	❍	❍	❍
4. Comprimidos de ferro devem ser consumidos por todos os atletas que se sentem cansados e estão pálidos **(D)**	❍	❍	❍

Q5.2 Todos os suplementos são testados para garantir que sejam seguros, que não tenham contaminação por substâncias proibidas.

- ❍ Concordo
- ❍ **Discordo**
- ❍ Não sei

Q5.3 Os rótulos dos suplementos às vezes podem dizer coisas que não são verdadeiras.

- ❍ **Concordo**
- ❍ Discordo
- ❍ Não sei

Q5.4 Você concorda com estas afirmações sobre suplementos?

	Concordo	Discordo	Não sei
1. A creatina traz a sensação que o exercício é mais fácil **(D)**	❍	❍	❍
2. A cafeína torna os músculos capazes de trabalhar mais, mesmo com pouco oxigênio **(D)**	❍	❍	❍
3. Suco de beterraba (nitratos) faz os músculos ficarem menos doloridos após o exercício **(D)**	❍	❍	❍
4. A beta-alanina pode diminuir a quantidade de ácido que os músculos produzem durante o exercício intenso **(C)**	❍	❍	❍

Q5.5 Qual suplemento não possui evidências suficientes em relação à melhora da composição corporal ou desempenho físico?

- ❍ cafeína
- ❍ **ácido ferúlico**
- ❍ bicarbonato de sódio
- ❍ creatina
- ❍ Não sei

Q5.6 A Agência Mundial Antidopagem (sigla em inglês, WADA) proíbe o uso de…

- ❍ cafeína
- ❍ bicarbonato de sódio
- ❍ carnitina
- ❍ **testosterona**
- ❍ Não sei

Bebida Alcoólica

Q6.1 Quanto de álcool contém em uma dose padrão de bebida alcoólica (ex. 1 dose de cachaça)?

- ❍ 1 – 2g de álcool por dose
- ❍ **8 - 14 g de álcool por dose**
- ❍ 30 - 50 g de álcool por dose
- ❍ Não sei

Q6.2 Das opções abaixo, qual é um exemplo de uma dose padrão de bebida alcoólica?

- ❍ **30 - 45 ml de destilado simples**
- ❍ Um quarto de garrafa (190ml) de vinho tinto
- ❍ Uma caneca grande (425 ml) de cerveja
- ❍ Não sei

Q6.1 Quanto de álcool contém em uma dose padrão de bebida alcoólica (ex. 1 dose de cachaça)?

- ❍ 1 – 2g de álcool por dose
- ❍ **8 - 14 g de álcool por dose**
- ❍ 30 - 50 g de álcool por dose
- ❍ Não sei

Q6.2 Das opções abaixo, qual é um exemplo de uma dose padrão de bebida alcoólica?

- ❍ **30 - 45 ml de destilado simples**
- ❍ Um quarto de garrafa (190ml) de vinho tinto
- ❍ Uma caneca grande (425 ml) de cerveja
- ❍ Não sei

Q6.3 Você acha que o álcool pode fazer você ganhar peso?

- ❍ **Sim**
- ❍ Não
- ❍ Não sei

Q6.4 Quantas doses de bebidas você acha que os especialistas recomendam como o máximo que devemos tomar em um dia?

- ❍ 1
- ❍ **2 - 3**
- ❍ 4 - 5
- ❍ Não sei

Q6.5 Você concorda com estas afirmações sobre o álcool?

	Concordo	Discordo	Não sei
1. Se alguém não bebe nada durante a semana, não há problema em tomar cinco ou mais drinques em uma única ocasião (sexta ou sábado à noite, por exemplo) **(D)**	❍	❍	❍
2. Beber muito álcool pode dificultar a recuperação de uma lesão **(C)**	❍	❍	❍
3. Álcool faz você urinar mais **(D)**	❍	❍	❍

Q6.6 "Beber pesado esporadicamente" é definido como:

- ❍ tomar duas a três bebidas alcoólicas padrão na mesma ocasião
- ❍ **tomar quatro a seis bebidas alcoólicas padrão na mesma ocasião**
- ❍ tomar sete ou mais bebidas alcoólicas padrão na mesma ocasião
- ❍ Não sei
